# Prevalence and predictors of depressive symptoms among married Egyptian women: a multicenter primary healthcare study

**DOI:** 10.1186/s12888-022-04239-w

**Published:** 2022-09-10

**Authors:** Doaa Mohamed Osman, Gellan K. Ahmed, Manal Mukhtar Farghal, Ahmed K. Ibrahim

**Affiliations:** 1 Public Health and Community Medicine Department, Faculty of Medicine, Assiut University, Assiut, Egypt; 2 Department of Neurology and Psychiatry, Faculty of Medicine, Assiut University, Assiut, Egypt; 3 Family Planning Physician, Egyptian Ministry of Health and Population, Cairo, Egypt

**Keywords:** Depressive symptoms, Women, Marital satisfaction, Egypt

## Abstract

**Background:**

Depression affects about 3.8% of the world’s population. Although marriage may contribute to subjective well-being, some marital variables could increase women’s risk for depression. This study aimed to determine the prevalence of depressive symptoms and their correlates among married females attending primary healthcare facilities.

**Methods:**

A cross-sectional study was conducted on a purposive sample of 371 married women at the primary healthcare centers, Assiut Governorate, Upper Egypt. In this study, an interviewer-administered questionnaire was used for data collection. Depressive symptoms were assessed using the Patient Health Questionnaire-9 (PHQ-9) and marital satisfaction using the ENRICH Marital Satisfaction Scale (EMS). Standardized measurements of weight and height were performed.

**Results:**

According to the PHQ-9 diagnostic criteria, the prevalence of depressive symptoms among the studied married females was 30.2%. The significant predictors of depressive symptoms were advanced husbands’ ages, living with an extended family, exposure to spousal verbal violence, high body weight, and low marital satisfaction levels.

**Conclusions:**

Approximately one-third of married Egyptian women experienced depressive symptoms. In addition to high body weight, some social and marital factors contributed to the increase in women’s vulnerability to depressive symptoms. Egyptian primary healthcare physicians should be trained to identify females with depressive symptoms and refer them to specialists if need be. To combat depression in women, it may be helpful to construct qualified marital counseling centers. This may improve marital satisfaction, decrease the negative consequences of spousal violence, and ensure the value of independence for new families.

## Background

In 2021, it was estimated that 3.8% of the global population had depression [1, 2]. More women are affected by depression than men [3], with an almost two-fold lifetime prevalence rate [4]. Depression is one of the leading causes of disability. It can be long-lasting or recurrent; it considerably impairs the individual’s performance at work or school and their ability to cope with daily life [5]. In the worst-case scenario, depression could lead to suicide [2].

Women have a 50% greater burden of depression and are more severely afflicted than men [6, 7]. This high prevalence of depression has been associated with hormonal fluctuations in females, especially during puberty, before menstruation, following pregnancy, and at perimenopause. It was proposed that women’s hormonal changes may be a predisposing factor for depression onset [8].

In subjective well-being research, human well-being is reported to be positively correlated with marriage [9]. However, some marital factors may increase women’s risk for depression [10]. The burdens of domestic work, children, in-laws, and marital stress may contribute to women’s higher depression rates than men’s [11]. In addition, spouse-related factors such as conflict with the husband, physical or non-physical partner violence, substance use behaviors, and lack of intimacy [12] are risk factors for depression in married women [13]. Furthermore, the low socioeconomic level is associated with depression among married Pakistani women. Low-income women often live in extended households where they must adjust to several relationships [14].

Depression is a complex phenomenon with no identifiable cause [15]. Beside marital factors, other factors that might increase the risk of depression include psychosocial stressors such as episodic stresses [16], family history of depression [12], obesity in western populations [17], lack of control at work, job pressure, the imbalance between effort and reward, and low professional social prestige might impair mental health [18]. Finally, a person’s mental health is likely affected by a long-term physical illness [19].

Mental diseases form a considerable proportion of morbidity seen in primary health centers [20]. Availability of mental services in primary healthcare means better accessibility to the needed care near patients’ homes, keeping their families together, and decreasing the indirect health expenditure associated with seeking care as transportation to mental health facilities located in big cities and loss of productivity due to the time spent in accompanying the mentally ill subject to specialized hospital [21].

The World Health Organization emphasized the value of integrating mental health care into primary care services and addressing the challenges of service delivery and accessibility as the associated stigma, low awareness, and chronicity [20].

Globally, the proportion of depression among females is 5.1%. The prevalence of depression varies by WHO Region [22]. About 10 percent of American women in the United States reported symptoms suggestive of experiencing a major depressive episode in the last year [23]. Depression prevalence was 7·74% among European women [24]. A systematic review found that the prevalence of perinatal depression in Asian women was 17% [25]. On the other hand, a Thai study found the prevalence of perinatal depression among women was 46.8% [26, 27].

In Egypt, prior work has reported that the Egyptian urban and rural populations had a lifetime prevalence of depression symptoms of 11.4% and 19.7%, respectively [28]. A cross-sectional study, depression was assessed using Beck’s Depression Inventory (BDI) among 568 Egyptian teachers (58.5% were females). According to this study, the prevalence of depression among female teachers was 28.3% [29].

A paucity of widescale surveys reports the prevalence of depression in the Egyptian population, especially among married women in upper Egypt. This is might due to cultural variables that impede the identification and treatment of depression, including younger marriage age, especially in rural regions [30], insufficient mental health care, illiteracy, early school dropout, unemployment, and the stigma of psychiatric diseases [31–33].

The current study aimed to determine the prevalence of depressive symptoms and their correlates among a sample of Egyptian married women attending primary healthcare centers in the Assiut Governorate, Upper Egypt.

## Methods

### Study site

Dairut district is one of the largest districts in the Assiut Governorate. It includes the city of Dairut and 41 villages. It is located in the north of the Assiut Governorate, Upper Egypt. According to the data from the Egyptian Ministry of Health on January 1, 2017, Dairut city has an estimated total population of 92,144 and the estimated number of families was 20,942. There are two primary healthcare facilities in Dairut city. One serves the Eastern part of the city and the other the Western part. The current study was conducted in primary healthcare centers in Dairut city, Assiut Governorate, Upper Egypt.

### Study design and population

A cross-sectional study was applied on 371 married females who attended the two primary health care centers for health care services such as vaccination, family planning, and antenatal care.

### Sample size and sampling technique

The sample size was calculated using EPI info version 7.2.4.0. Based on an expected frequency of 22.4% [34], an acceptable margin of error of 5%, a design effect of 1, and a confidence interval of 97%, the minimum required sample was 327 married females. Non-probability purposive sampling technique was used.

### Data collection

Data collection was carried out from July 2018 to December 2018. Data were collected using interviewer-administered semi-structured questionnaires, and anthropometric measurements were also recorded. The questionnaires included:

1. Sociodemographic data of women and their husbands such as age, residence, educational level, occupation, and whether husband has other wives.
2. Self-reported health status and obstetric history, including self-reported chronic diseases (cardiac diseases, diabetes, and hypertension), history of mental illness, and self-satisfaction with body weight. The questionnaire also had questions on parity, stillbirth, abortion, current pregnancy, number and sexes of living children, and the presence of congenital anomalies or disabilities in children [35–38].
3. Family and marital variables such as type of family (extended or nuclear), number of family members, the occurrence of death in the family during the last three months, family history of mental illness, age of first marriage, and marriage duration [39, 40].
4. Exposure to spousal violence included both physical and verbal spousal violence, being terrified by one’s husband, and having an addict husband [41, 42].
5. Family wealth was measured using the Family Affluence Scale (FAS). The concept of the FAS is related to common material deprivation and home affluence consumption indices. The FAS inquiries about four items (cars, bedrooms, vacations, and computers). Based on the replies to all four, a composite score was calculated for each woman. A score of ≤ 2 or less indicates low affluence, a score of 3 to 5 indicates medium affluence, and a score of ≥ 6 indicates high affluence [43].
6. Marital satisfaction was assessed using the ENRICH marital satisfaction Scale (EMS): The EMS Scale is an instrument used to briefly assess marital quality (evaluation and nurturing issues, communication, and happiness) [44]. It is a fifteen-item scale with two subscales: five of its items evaluate idealistic distortion and ten assess marital satisfaction. There are five response options for each item (strongly disagree, moderately disagree, neither agree nor disagree, moderately agree, and strongly agree). Each item may be scored positively or negatively based on its sign described in the scale. Positive items are valued from strongly disagree (1) to strongly agree (5). Negative items are scored in the opposite direction to positive ones, with (3) standing for neither agree nor disagree in both cases.
  After calculating the raw scores, the corresponding percentile scores were obtained from the norm table in the EMS scale guide. Individual EMS scores are obtained using this following formula: EMS score = PCT–[(.40 ×PCT) (ID × .01)]; where PCT = percentile score for individual marital satisfaction scale and ID = percentile score for the individual idealistic distortion scale [44].
7. The Patient Health Questionnaire-9 (PHQ-9) is a nine-item instrument used to make criteria-based diagnoses for depressive and other primary care mental disorders [45]. It asked about the frequency of certain problems over the last two weeks. Each item has four response options that range from not at all (0) to nearly every day (3).
  The researchers added the values for each response in the questionnaire to get the total PHQ-9 score. The PHQ-9 guide was used for interpreting the score. Its interpretation was as follows: normal range = 0–4, minimal depression symptoms = 5–9, major depression symptoms with a mild degree = 10–14, major depression symptoms with a moderate degree = 15–19, major depression symptoms with a severe degree = ≥20). The presence of major symptoms of depression was taken for a PHQ-9 score of ≥10. The studied women who had a score PHQ-9 score less than 10 were considered as non-major depressive symptom category [39, 46, 47].
8. Measurement of some anthropometric parameters: the weight and height were measured following standard methods. The body mass index (BMI) was calculated as the weight in kilograms divided by the square of the height in meters. Twenty-three women were excluded because of their body weight and BMI values (since they were pregnant). Weight categories were determined based on the International Classification of adults. The different categories were underweight, normal weight, overweight, and obesity according to each person’s BMI [48].

### Statistical analysis

Data analysis was performed using IBM-SPSS version 21 [49]. Qualitative data were expressed as frequencies and percentages while quantitative data were expressed as the mean ± standard deviation. The reliability of the used scales was assessed. The calculated Cronbach’s alpha coefficients for the used scales were as follows: 0.845 for PHQ-9, 0.339 for the FAS scale, and 0.867 for the ENRICH scale. The outcome variable was the presence of depressive symptoms (yes = 1, and no = 0). The explanatory variables were constructed after reviewing the available medical literature. Basic bivariate analyses were conducted to test the associations between depressive symptoms and the explanatory variables before multivariate analyses to explore the most important risk factors. Multivariate logistic backward stepwise regression model was performed; the authors included in the model all the significant variables from the bivariate screening analyses. The initial and final models were presented in Table 4. Adjusted odds ratios and 95% confidence intervals were reported for the constructed models. For all statistical tests, *p*-values of less than 0.05 were considered statistically significant. Graphic presentation of data was done using Microsoft Excel.

## Results

**Fig. 1:**
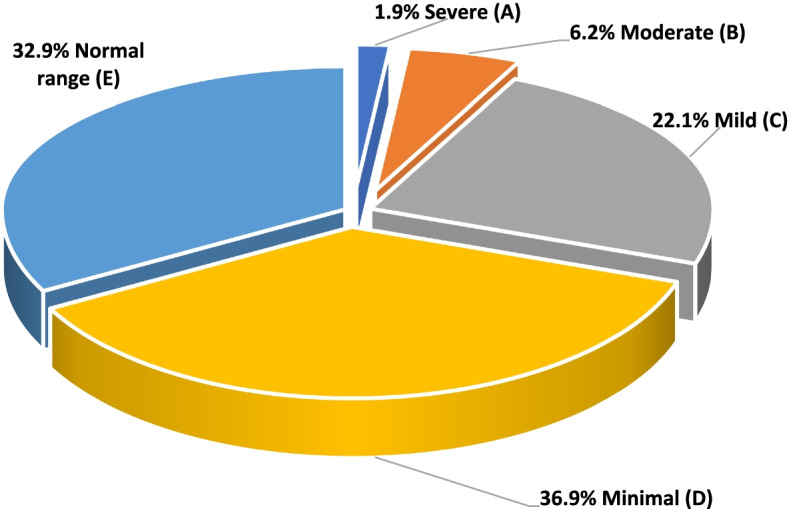
Distribution of depressive symptoms’ grades using the PHQ-9 among studied Egyptian married women

According to the PHQ-9, 32.9% of females were in the normal range, 36.9% had mild depression symptoms, and only 1.9% had severe depression symptoms. Based on PHQ-9 guidelines, 30.2% of the participants had depression symptoms while 69.1% of them did not have (See Fig. 1). The PHQ-9 score among studied women ranged from 0 to 27 with a mean value of 7.35 ± 4.8.

The sociodemographic data and depressive symptom associates were presented in Table 1. The ages of the study participants ranged from 18 to 59 years with a mean value of 32.35 ± 7.62 years. Depressed females had higher mean ages and body weights compared to non-depressed women (*P*-value = 0.029 and 0.023, respectively). Also, the mean age of the participants’ husbands was higher among depressed women than among non-depressed ones (*P*-value = 0.015). On the other hand, there was no statistically significant association between all sociodemographic characteristics and depressive symptoms.

**Table 1:** Personal characteristics of studied Egyptian married females and their husbands

	Total (no = 371)	Depressive symptom (*n* = 112)	No Depressive symptom (*n* = 259)	*P* value
Age in years				
• < 30	148(39.9%)	34(23.0%)	114(77.0%)	
• 30—< 40	158(42.6%)	56(35.4%)	102(64.6%)	0.046*
• ≥ 40	65(17.5%)	22(33.8%)	43(66.2%)	
Mean ± SD	32.35 ± 7.62	33.68 ± 7.65	31.78 ± 7.55	0.029*
Residence:				
• Urban	171(46.1%)	50(29.2%)	121(70.8%)	
• Rural	200(53.9%)	62(31.0%)	138(69.0%)	0.713
Women educational level				
• Illiterate/ Reads & writes	87(23.5%)	33(37.9%)	54(62.1%)	
• Basic education	48(12.9%)	11(22.9%)	37(77.1%)	
• Secondary/ Technical institute	160(43.1%)	48(30.0%)	112(70.0%)	0.238
• University/ Postgraduate	76(20.5%)	20(26.3%)	56(73.7%)	
Female occupation:				
• Housewife	274(73.9%)	85(31.0%)	189(69.0%)	0.557
• Working	97(26.1%)	27(27.8%)	70(72.2%)	
Weight categories (*n* = 348^a^)				
• Underweight / Normal	109(31.3%)	30(27.5%)	79(72.5%)	
• Overweight	124(35.6%)	31(25.0%)	93(75.0%)	0.375
• Obese	115(33.0%)	38(33.0%)	77(67.0%)	
Body mass index (Mean ± SD)	27.95 ± 5.29	28.64 ± 5.67	27.68 ± 5.11	0.125
Weight (Mean ± SD)	71.70±14.03	74.40±15.82	70.62±13.14	0.023*
Husband age / years Mean ± SD (Range)	38.2 ± 9	39.9 ± 9.4	37.4 ± 8.7	0.015*
Husband educational level				
• Illiterate/ Reads & writes	74(19.9%)	25(33.8%)	49(66.2%)	
• Basic education	22(5.9%)	7(31.8%)	15(68.2%)	0.859
• Secondary/ Technical institute	200(53.9%)	57(28.5%)	143(71.5%)	
• University/ Postgraduate	75(20.2%)	23(30.7%)	52(69.3%)	
Husband occupation				
• Employee	113(30.5%)	34(30.1%)	79(69.9%)	
• Farmer	36(9.7%)	14(38.9%)	22(61.1%)	
• Skilled worker	78(21.0%)	24(30.8%)	54(69.2%)	0.243
• Unskilled worker	57(15.4%)	14(38.9%)	22(61.1%)	
• Free business	74(19.9%)	16(28.1%)	41(71.9%)	
• Not working	13(3.5%)	17(23.0%)	57(77.0%)	
Your current husband has other wives				
• Yes	20(5.4%)	8(40%)	12(60%)	0.326
• No	351(94.6%)	104(29.6%	247(70.4%)	

^a^23 cases were excluded due to pregnancy, *SD* Standard deviation, *significant *p* value

As illustrated in Table 2, the type of family and the occurrence of death in the last three months were associated with the presence of depressive symptoms. A family history of mental illness, socioeconomic status, women’s age at marriage, and marriage duration were not significantly associated with depressive symptoms. Husbands’ physical violence was experienced by 27% of our study participants. Similarly, approximately 28% of them reported exposure to verbal violence. Husbands’ addiction status was reported by 10% of our participants. Approximately 43% of them were afraid of their current husbands. Depressive symptoms had higher percentage of exposure to spousal physical or verbal violence, husbands’ addiction, and participants’ fear of their husbands (*P*-value < 0.05). Depressed women had lower mean marital satisfaction scores than non-depressed women (*P*-value < 0.001).

**Table 2:** Family characteristics, marital variables, Socioeconomic levels, and marital satisfaction scale of the studied Egyptian married women

	Total (no = 371)	Depressive symptom (*n* = 112)	No Depressive symptom (*n* = 259)	*P* value
Type of family				
• Nuclear	259(69.8%)	70(27.0%)	189(73.0%)	0.044*
• Extended	112(30.2%)	42(37.5%)	70(62.5%)	
No. of family members:				
• ≤ 5	151(40.7%)	39(25.8%)	112(74.2%)	0. 130
• > 5	220(59.3%)	73(33.2%)	147(66.8%)	
• Mean + SD	6.93 ± 3.93	7.09 ± 3.33	6.86 ± 4.17	0.608
Socioeconomic levels using Family Affluence Scale (FAS)				
• low	271(73.0%)	88(32.5%)	183(67.5%)	0.195
• Middle	91(24.5%)	23(25.3%)	68(74.7%)	
• High	9(2.4%)	1(11.1%)	8(88.9%)	
Mean of FAS ± SD	7.35 ± 4.84	1.80 ± 1.146	2.07 ± 1.43	0.055
Family history of mental illness ^a^				
• Yes	12(3.2%)	6(50.0%)	6(50.0%)	0.197
• No	359(96.8%)	106(29.5%)	253(70.5%)	
Death in the family during the last 3 months				
• Yes	45(12.1%)	20(44.4%)	25(55.6%)	0.026*
• No	326(87.9%)	92(28.2%)	234(71.8%)	
Woman age at first marriage: (years) Mean ± SD (Range)	20.4 ± 4.1(12—37)	20.5 ± 4	20.3 ± 4.1	0.575
Duration of current marriage:(years) Mean ± SD (Range)	12 ± 8.1 (1—40)	13.1 ± 7.6	11.5 ± 8.3	0.074
Ever exposed to Physical violence				
• Yes	100(27%)	44(44%)	56(56%)	0.001*
• No	271(73%)	68(25.1%	203(74.9%)	
Ever exposed to Verbal violence				
• Yes	105(28.3%)	47(44.8%)	58(55.2%)	0.001*
• No	266(71.7%)	65(24.4%)	201(75.6%)	
Current husband drinking alcohol or taking drugs				
• Yes	37(10%)	17(45.9%)	20(54.1%)	0.028*
• No	334(90%)	95(28.4%)	239(71.6%)	
Fear of current husband				
• Yes	159(42.9%)	59(37.1%)	100(62.9%)	0.012*
• No	212(57.1%)	53(25%)	159(75%)	
Marital satisfaction (ENRICH marital satisfaction Scale) (EMS)				
Mean ± SD	45.85 ± 13.16	41.1 ± 17	47.9 ± 10.5	< 0.001*

^a^Fisher Exact test, *FAS* Family Affluence Scale, *EMS* The ENRICH marital satisfaction Scale, *SD* Standard deviation, *significant *p* value

Table 3 shows the association between depressive symptoms, participants’ self-reported health statuses, and obstetric history of married females. Depressive symptoms had higher percentage of the presence of chronic diseases and ongoing pregnancies. On other hand, depressive symptoms were not significantly associated with mental illness, perceived satisfaction with one’s body weight, number of pregnancies, the number and sexes of living children, having children with congenital anomalies or disabilities, and a history of stillbirth or abortion (*P* > 0.05).

**Table 3:** Self-reported health status, and obstetric history of studied of the studied Egyptian married women

	Total (no = 371)	Depressive symptom (*n* = 112)	No Depressive symptom (*n* = 259)	*P* value
Chronic disease:				0.036*
• Yes	35(9.4%)	16(45.7%)	19(54.3%)	
• No	336(90.6%)	96(28.6%)	240(71.4%)	
Mental illness ^a^				
• Yes	15(4.0%)	6(40.0%)	9(60.0%)	0.399
• No	356(96.0%)	106(29.8%)	250(70.2%)	
Weight perception				
• Satisfied	263(70.9%)	77(29.3%)	186(70.7%)	0.551
• Dissatisfied	108(29.1%)	35(32.4%)	73(67.6%)	
No. of pregnancies				
• < 3	96(25.9%)	22(22.9%)	74(77.1%)	0.071
• ≥ 3	275(74.1%)	90(32.7%)	185(67.3%)	
Living children				
• Males	53(14.3%)	17(32.1%)	36(67.9%)	0.405
• Females	38(10.2%)	11(28.9%)	27(71.1%)	
• Both	268(72.2%)	83(31.0%)	185(69.0%)	
• No children	12(3.2%)	1(8.3%)	11(91.7%)	
Stillbirth				
• Yes	43(11.6%)	17(39.5%)	26(60.5%)	0.156
• No	328(88.4%)	95(29.0%)	233(71.0%)	
No. of abortions				
• Zero	114(30.7%)	39(34.2%)	75(65.8%)	0.261
• One or more	257(69.3%)	73(28.4%)	184(71.6%)	
Child with congenital anomalies /disabled				
• Yes	18(4.9%)	6(33.3%)	12(66.7%)	0.766
• No	353(95.1%)	106(30.0%)	247(70.0%)	
Currently pregnant				
• Yes	23(6.2%)	13(56.5%)	10(43.5%)	0.005*
• No	348(93.8%)	99(28.4%)	249(71.6%)	

^a^Fisher Exact test, *significant *p* value

Table 4 shows our study participants’ multivariable regression models for depressive symptom risk factors. The final model included five predictors. Women’s weight and husbands’ age were positive predictors of depression symptoms among married females (OR = 1.025, 95%CI: 1.006—1.044, OR = 1.030, 95%CI: 1.002—1.059 respectively). Low marital satisfaction scores were likely to increase the risk of women’s depressive symptoms (OR = 0.965, 95%CI: 0.946—0.984). Women’s exposure to spousal verbal violence were more likely to duplicates the risk for the depressive symptom (OR = 2.00, 95%CI: 1.151- 3.496). Women living with an extended family were more vulnerable to develop depressive symptoms than those living in nuclear families (OR = 1.956, 95% CI: 1.130—3.387).

**Table 4:** Multivariate logistic regression analysis of depressive symptoms among studied Egyptian married women

	**Initial model**				**Final model**			
	**OR**	**95% C.I**		***P*-value**	**OR**	**95% C.I**		***P*-value**
		**Lower**	**Upper**			**Upper**	**Lower**	
**Age in years**	0.982	0.927	1.041	0.547				
**Weight-kg** ^a^	1.024	1.005	1.043	0.013	1.025	1.006	1.044	0.009
**Chronic disease (yes)**	1.361	0.587	3.152	0.473				
**Death in family (yes)**	1.389	0.665	2.905	0.382				
**Husband age**	1.039	0.989	1.092	0.126	1.030	1.002	1.059	0.037
**Exposure to spousal physical violence (yes)**	1.270	0.622	2.593	0.511				
**Exposure to spousal verbal violence (yes)**	1.673	0.808	3.464	0.166	2.006	1.151	3.496	0.014
**Husband addiction (yes)**	0.878	0.381	2.023	0.760				
**Fear from husband (yes)**	1.129	0.644	1.981	0.671				
**Family type (extended)**	1.820	1.040	3.185	0.036	1.956	1.130	3.387	0.017
**Marital satisfaction (EMS score)**	0.966	0.946	0.985	0.001	0.965	0.946	0.984	0.001

Reference group in categorical variables: chronic disease (no), death in family (no), exposure to spousal physical violence (no), exposure to spousal verbal violence (no), husband addiction (no), fear from husband (no) and family type (nuclear)

Backward stepwise regression analysis was applied

^a^23 cases were excluded in the model due to their pregnancy, *significant *p* value

## Discussion

Depressive symptomatology is one of the most frequent mental health difficulties among women [39]. It is associated with functional disability, poor quality of life, the deterioration of comorbid physical diseases, and a higher risk of developing other chronic diseases [50]. In this study, we investigated the prevalence of depressive symptoms and their predictors among Egyptian married women.

According to the PHQ-9 diagnostic criteria, the prevalence of depressive symptoms was 30% in the present study among married Egyptian females attending healthcare centers. A similar prevalence of depression symptoms (28.3%) was reported among Egyptian female teachers using the BDI [29]. However, a lower prevalence was reported by Okasha A (1999) where the Egyptian urban and rural populations had a lifetime prevalence of depression symptoms of 11.4% and 19.7%, respectively [28]. This higher prevalence of depression symptoms in the present study could be explained by the fact that our study participants were all females who are more susceptible to depression symptoms [8]. Moreover, there is growing evidence that depression symptoms is a public health problem of increasing magnitude [51].

The prevalence of depression symptoms in different countries and societies has been reported to vary greatly. Also, predictors of depression symptoms differ greatly in different cultural settings [52]. This could be explained by the variations in age groups between the different study populations, the presence of comorbid illnesses, depression symptoms measurement tools or cut-off points used to define depression symptoms, and the prevailing economic situations of the different countries. In the World Mental Health Survey, a higher prevalence of major depressive symptoms was reported in developing countries (except for East/Southeast Asia) than in developed countries [53].

Compared to the current study, higher prevalence rates of depression symptoms were reported in Bangladesh, Ghana, and Brazil might be attributed to the underlying characteristics of the studied women. A higher prevalence rate (65%) was detected among Bangladeshi women aged ≥ 18 years who survived cyclone mora in 2017. This is explained by the fact that they live in disaster-prone areas, which predisposes them to mental health problems [54]. The prevalence of depression symptoms among infertile Ghanaian women was 62.0%, which is higher than the prevalence in this study. This higher prevalence could be explained by the fact that childless African women face a lot of societal discrimination, which could result in psychological disorders [55]. In Brazilian middle-aged women, the prevalence of depressive symptoms was 45.7%, which is higher than the prevalence we found in this study. This higher prevalence could be explained by the fact that all the participants of this study were premenopausal women [56].

The study site may have contributed to the overestimation of the prevalence of depression symptoms in some studied population. A Pakistani hospital-based study reported a higher prevalence of depression symptoms (62%) among women exposed to violence who sought care from a psychiatric hospital. This high prevalence could be attributed to the sampling method that targeted the women who sought mental health care from a specialized psychiatric center as well as the spousal experience of violence [41].

A lower prevalence of depression symptoms was reported in more developed countries, including Italy, (26%), Sweden (22.4%), South Africa (20.5%), Korea (9.1%), Croatia (25.5%), and rural China (12.4%) [34, 57–61]. However, lower prevalence rates were reported in developing countries, such as 24.2% among Indian women, although they were middle-aged females with many premenopausal symptoms [39]. In Mozambique (2014), 14% of females were diagnosed with depression symptoms [36].

In our study, age was significantly associated with depressive symptoms. However, age was not a significant predictor of depression symptoms among married women after the adjusted regression analysis (OR = 0.982, 95% CI:0.927, 1.041). similarly, A cross-sectional study of Turkish women found no statistically significant association between the presence of depressive symptoms in women and their ages [62].

In contrast, the prevalence of depression symptoms was significantly associated with advanced age in many other studies that explored correlates of depression symptoms. The prevalence of depression symptoms among Pakistani married women increased by 1.8% for every one-year increase in age [63]. The baseline age was found to be significantly associated with major depression symptoms among African and Caucasian American women in the Mental Health Study of Women’s Health Across the Nation [64]. Also, self-reported depression symptoms were more common among older females, according to a secondary analysis of the fifth Korean National Health and Nutrition Examination Survey [58].

In the present study, an adjusted regression model revealed that high body weight was a significant predictor of depressive symptoms among the study participants. Similar results were reported upon studying the relationship between obesity and depression symptoms in Egyptian women who attended the Obesity Clinic at the National Research Center. The prevalence of depression symptoms was significantly higher in obese women than in non-obese ones [65]. The BMI was significantly associated with major depression symptoms among African and Caucasian American women in the Women’s Health across the Nations study (OR = 1.07, 95% CI: 1.03–1.11) [64]. On the other hand, depressive symptoms were more prevalent in underweight and normal weight women than in overweight/obese women in rural India [39].

Participants’ husband’s age was a significant predictor of depressive symptoms (OR = 1.030, 95% CI: 1.002,1.059). Similarly, Indian women with age differences of more than ten years between themselves and their husbands were found to be more depressed [66].

In this study, there was no statistically significant difference in the mean socioeconomic score between women have depressive symptoms and women without depressive. Similarly, no such association was found in a study conducted among rural Indian middle-aged women according to which depression symptoms affects both rich and poor people equally [39].

However, several studies have reported that poverty and low socioeconomic status are associated with depression symptoms. Poor people perceive themselves as socioeconomically disadvantaged and relatively deprived compared to others. This may result in frustration, shame, feelings of inferiority, and stress, which in turn could have adverse impacts on health, including depression symptoms [67]. Among Brazilian women, depressive symptoms were more prevalent in those belonging to the first and second economic index categories than in those of the highest category [68]. The data of married females from the 8^th^ wave of the Korea Welfare Panel Study and from the fifth Korean National Health and Nutrition Examination Survey among Korean working women were analyzed. Both studies found that depressive symptoms were significantly associated with low household income [58, 69]. In the German Health Update study, a lower objective socioeconomic level was significantly associated with a higher risk of current depressive symptoms in German women [67].

Many women experience mental health issues after major negative life events such as the death of a loved one [63]. In the present study, the adjusted regression model revealed no significant of the occurrence of death in the family during the last three months for depression symptoms (OR:1.389,95%CI:0.665,2.005). However, a recent death in the family was associated with an increased risk of developing depression symptoms among married women in Pakistan [63]. Extend families are more prevalent than nuclear families in the Arab world, particularly in Egypt. This offers replacements for lost or absent parental figures, conflict mediation, preferred hiring of family members, and assistance with healthcare costs. In Arab nations, families are responsible for the prevention, treatment, and aftercare of illness and death [70, 71].

In the present study, depressive symptoms were significantly associated with exposure to physical or verbal spousal violence. After adjusted analyses, experiencing verbal violence from spouses was a significant predictor of depressive symptoms among married women. Similarly, Park et al. found that Korean women who experienced both non-physical and physical intimate partner violence were more likely to report depressive symptoms [69].

History of intimate partner violence was found to be an independent predictor of depression symptoms (OR = 6.07, 95%CI:2.8,12.7) among rural Indian women [39]. South African women with depressive symptoms reported significantly more incidents of physical or sexual intimate partner violence (OR = 2.21, 95%CI 1.16, 3.00) [60]. Abuse by spouses or in-laws contributed significantly to depression symptoms among Pakistani married women (OR = 5.21; CI = 2.79, 9.42) [72].

According to the results of the present study, there was a significant association between depressive symptoms and current alcohol/drug consumption by the participants’ husbands. However, this was not a significant predictor of depressive symptoms among our study participants (OR = 0.878,95% CI:0.381, 2.023).

Variations in the association between alcohol/substance abuse and depressive symptoms were reported. Indian women who stayed in families with substance abuse issues [66] and whose spouses were alcoholics (OR: 5.84, 95%CI: 2.8, 12.2) had significantly higher odds of developing depression symptoms [39]. However, on analyzing the data of 4659 married females from the Korea Welfare Panel Study’s 8^th^ wave, it was seen that having partners who were reported as heavy drinkers was not associated with depressive symptoms (OR: 1.29, 95% CI: 0.74, 2.27) [69].

Regarding extended families, when comparing women who lived with extended families to those who lived in single families, the odds ratio of depressive symptoms was higher among extended family women (OR = 1.82, 95% CI: 1.04, 3.18). Also, women living with joint families were found to be significantly more depressed in a cross-sectional study conducted among ever-married women in India [66]. Another study conducted in India found that the prevalence of major depression symptoms among women who lived with extended families compared to women in the nuclear family was significantly greater [39].

On the contrary, the family system significantly affects the level of depression symptoms among working married women. It was discovered that working women in Pakistan who lived with nuclear families were more depressed than those who lived with joint families. This is because, in a joint family system, relatives may be able to assist working mothers with the child and household duties. In contrast, non-working women living in nuclear family systems have no significant difference in their levels of depression symptoms because they must raise their children as well as do all household chores by themselves [73].

As stated by the Marital Discord Model of depression symptoms, marital discord is a crucial predictor of depression symptoms in a large proportion of married people [74]. Reduced marital satisfaction has been associated with an increased rate of concurrent depression symptoms and a rise in the risk of future depression symptoms. Among Chinese married women, relationship satisfaction was a significant negative predictor of depression symptoms [75]. In the current study, the mean marital satisfaction score was significantly lower among depressed females than among non-depressed ones. Furthermore, in a multivariate regression model, high marital satisfaction was a significant negative correlate of depressive symptoms among currently married females.

### Limitations and recommendations of the study

The current study is insufficient to provide a complete representative picture of all Egyptian married women due to the applied non-probability sampling technique. Moreover, it was conducted only among women in Upper Egypt who sought primary healthcare services. Also, some study participants had to be excluded from the adjusted analysis to evaluate the effect of weight on depression status because they were pregnant. Lastly, this study’s Cronbach’s alpha coefficients for FAS were low. Although FAS has been widely used in research recently to reflect the family affluence replacing income, which is sensitive to people, it is less used in low/middle-income countries. We reckon the low reliability is caused by the socioeconomic differences between high and low/middle-income countries. Thus, it needs modifications to suit these populations. The authors used several props to investigate the SES of the targeted population (women and husbands’ education/occupation and residence); we assume it does not affect the overall relationship between SES and depressive symptoms.

Based on the findings of this study, we recommend that primary healthcare physicians should be trained to identify depressed females and refer them to specialists for psychological support and appropriate management. To reduce the prevalence of depression symptoms in women, it may be helpful to raise social awareness through mass media campaigns about the negative consequences of spousal violence on women’s mental health and the value of the independence of new families. In addition, improving marital satisfaction through the construction of qualified marital counseling centers (state-owned and private organizations) that are already deficient in the Egyptian community may contribute to reducing the prevalence of depression symptoms among married women.

## Conclusion

In the present study, the prevalence of depressive symptoms was 30.2%. Among currently married females, there were five correlates that were significantly identified as predictors of depressive symptoms, which included advanced husbands’ ages, high body weights, extended family types, exposure to verbal violence by the husband, and low marital satisfaction scores.

## Data Availability

All data generated or analyzed during this study are available from corresponded on request.

## Abbreviations

PHQ-9: Patient Health Questionnaire-9
FAS: Family Affluence Scale
EMS: ENRICH Marital Satisfaction Scale
BMI: Body mass index
